# Effects of *CYP3A5* polymorphism on the pharmacokinetics of a once-daily modified-release tacrolimus formulation and acute kidney injury in hematopoietic stem cell transplantation

**DOI:** 10.1007/s00280-016-3060-4

**Published:** 2016-05-23

**Authors:** Takaya Yamashita, Naohito Fujishima, Masatomo Miura, Takenori Niioka, Maiko Abumiya, Yoshinori Shinohara, Kumi Ubukawa, Miho Nara, Masumi Fujishima, Yoshihiro Kameoka, Hiroyuki Tagawa, Makoto Hirokawa, Naoto Takahashi

**Affiliations:** Department of Hematology, Nephrology and Rheumatology, Akita University Graduate School of Medicine, Akita, Japan; Division of Blood Transfusion, Akita University Hospital, 1-1-1 Hondo, Akita, 010-8543 Japan; Department of Pharmacy, Akita University Hospital, Akita, Japan; Department of General Internal Medicine and Clinical Laboratory Medicine, Akita University Graduate School of Medicine, Akita, Japan

**Keywords:** Once-daily tacrolimus formulation, *CYP3A5* polymorphism, Azole antifungal agent, Pharmacokinetics, Hematopoietic stem cell transplantation

## Abstract

**Background:**

Tacrolimus is metabolized by cytochrome P450 (CYP) 3A4 and 3A5. We investigated the influence of *CYP3A5* polymorphism and concurrent use of azole antifungal agents (AZ) on the pharmacokinetics of a once-daily modified-release tacrolimus formulation (Tac-QD) in patients after hematopoietic stem cell transplantation (HSCT).

**Design and methods:**

Twenty-four patients receiving allogeneic HSCT were enrolled. Genotyping for CYP3A5*3 was done by a PCR-restriction fragment length polymorphism method. Trough blood concentrations (C_0_) of tacrolimus were measured by chemiluminescence magnetic microparticle immunoassay. Continuous infusion of tacrolimus was administered from the day before transplantation and was switched to Tac-QD after adequate oral intake.

**Results:**

Thirteen patients had a CYP3A5*3/*3 genotype, and 11 patients had a CYP3A5*1/*1 or *1/*3 genotype. No significant difference was observed in daily dosages and the C_0_ of tacrolimus between the two genotype groups without AZ. However, in patients who were co-administered AZ, the C_0_ values of tacrolimus were higher in patients with the CYP3A5*3/*3 allele than with the CYP3A5*1 allele (*P* = 0.034), although daily doses of Tac-QD in patients with CYP3A5*3/*3 were significantly lower than those with the CYP3A5*1 allele (*P* = 0.041). The cumulative incidence of acute kidney injury was higher in patients with the CYP3A5*3/*3 than with the CYP3A5*1 allele when AZ was co-administered. The decrement for daily dosage of Tac-QD was significantly greater in patients expressing the CYP3A5*3/*3 than the CYP3A5*1 allele.

**Conclusions:**

*CYP3A5* genotyping may be useful for safe and effective immunosuppressive therapy with Tac-QD in HSCT patients in whom the use of AZ is anticipated.

**Electronic supplementary material:**

The online version of this article (doi:10.1007/s00280-016-3060-4) contains supplementary material, which is available to authorized users.

## Introduction

Tacrolimus has been widely used to prevent graft rejection following solid organ transplantation and hematopoietic stem cell transplantation (HSCT). Tacrolimus plus methotrexate is regarded as one of the standard methods of graft-versus-host disease (GVHD) prophylaxis after allogeneic HSCT. Given that tacrolimus has a narrow therapeutic window and that there are inter- and intra-individual variations in pharmacokinetics, blood concentrations of tacrolimus are usually monitored to maintain adequate exposure and to prevent drug-related toxicities [1].

Tacrolimus is given intravenously in the early phase after HSCT and then switched to oral administration. Oral tacrolimus was first developed as a twice-daily (BID: bis in die) formulation (Tac-BID). Then, a once-daily modified-release (QD: quaque die) formulation of tacrolimus (Tac-QD) was developed to provide more convenient dosing and to improve patient adherence [2]. In solid organ transplantation, initial clinical trials showed that the pharmacokinetic parameters of Tac-BID and Tac-QD were equivalent on a mg for mg basis, and Tac-QD was well tolerated with similar efficacy and safety profiles to Tac-BID [3–6]. However, studies of the pharmacokinetics of Tac-QD administration in the HSCT setting are few in number compared with solid organ transplantation. Thus, the pharmacokinetics of inter-individual variations of blood concentration of tacrolimus in transplant patients with Tac-QD after HSCT are yet to be determined.

Many clinical and genetic factors affect the pharmacokinetics of tacrolimus [7]. Cytochrome P450 (CYP) 3A4, CYP3A5 and ATP-binding cassette subfamily B member 1 (ABCB1) reportedly contribute to inter-individual variability in the absorption, metabolism and tissue distribution of tacrolimus. Moreover, CYP3A5 may play a dominant role over that of CYP3A4 in the metabolism of tacrolimus in individuals who express the CYP3A5 enzyme [8–12]. CYP3A5 is polymorphically expressed, and more than 10 single nucleotide polymorphisms (SNPs) have been identified [9]. The most important SNP related to functional variation is *CYP3A5* 6986A > G, in which the wild-type allele is CYP3A5*1 and the variant allele is CYP3A5*3 [13]. Homozygous carriers of the CYP3A5*3 gene (CYP3A5*3/*3) should lack functional CYP3A5 protein [14–18]. There are racial differences in the frequencies of *CYP3A5* polymorphisms [15, 19–21]. The frequencies of CYP3A5*3/*3 were reported to be 56.7–60.5 % in Japanese, 86 % in Caucasians and 23 % in the African-American population. Although there are many genetic variants of CYP3A4 and ABCB1, the majority of studies have failed to find an association between the CYP3A4 or ABCB1 genotypes and tacrolimus pharmacokinetics [9]. These studies have demonstrated that trough blood concentrations or area under the blood concentration–time curve (AUC) of tacrolimus was higher in patients with the CYP3A5*3/*3 genotype than those with the CYP3A5*1 allele, and the required daily dosage of tacrolimus was significantly reduced.

Our colleagues have previously reported the impact of *CYP3A5* polymorphism on the pharmacokinetics of tacrolimus in kidney transplantation patients receiving Tac-QD administration [22–25]. However, the effect of the *CYP3A5* genotype on the pharmacokinetics of Tac-QD in HSCT patients has yet to be clarified [13]. Although azole antifungal agents (AZ) are most often used for the prevention as well as the treatment of these infections in HSCT patients, these agents interfere with the metabolism and transport of tacrolimus [26]. In addition, the magnitude of drug interactions between AZs and tacrolimus differs between AZs (itraconazole = voriconazole > fluconazole) [27, 28]. Here, we investigated the safety and efficacy of Tac-QD in HSCT patients. We focused on *CYP3A5* polymorphism and the interaction of Tac-QD with AZ.

## Methods

### Recipients and protocols of transplantation

This study was conducted as a single-institution, prospective cohort study to evaluate the safety and efficacy of Tac-QD in allogeneic HSCT patients. The eligibility criteria for the present study were as follows: (1) HSCT patients were given the same immunosuppressive regimens for the prophylaxis of GVHD; (2) age > 16 years; (3) no hypersensitivity to tacrolimus (Prograf^®^ or Graceptor^®^); (4) no liver dysfunction [aspartate aminotransferase (AST) or alanine aminotransferase (ALT) level < fivefold the upper normal range and total bilirubin level < 2.0 mg/dL]; (5) no renal dysfunction (serum creatinine level < 2.0 mg/dL or creatinine clearance > 30 mL/min); and (6) no consumption of drugs or food affecting CYP3A and ABCB1 function. Twenty-four patients (17 males and 7 females) who underwent allogeneic HSCT at Akita University Hospital between April 2012 and October 2014 were enrolled in this study.

Prophylaxis regimens against GVHD included tacrolimus and methotrexate in the majority of patients, or variations including mycophenolate mofetil. Continuous infusion of 0.03 mg/kg/day tacrolimus was administered from the day prior to stem cell infusion. Tacrolimus administration was converted from intravenous administration to once-daily oral intake after adequate oral intake was observed. A quadruple dose of intravenous daily dose as an initial Tac-QD was administrated orally. The target whole blood concentrations of tacrolimus were 10–20 ng/mL with continuous infusion and 5–10 ng/mL after switching to oral administration. The whole blood concentrations of tacrolimus were measured by chemiluminescence magnetic microparticle immunoassay (CMIA).

Intravenous infusion of micafungin was given to prevent fungal infection during the neutropenic phase. Seven days after switching from tacrolimus continuous infusion to Tac-QD, antifungal agents were switched from intravenous micafungin to oral AZ.

This study was approved by the Ethics Committee of the Akita University School of Medicine, and each patient provided written informed consent in accordance with the Declaration of Helsinki.

### Genotyping

DNA was extracted from a peripheral blood sample using the QIAamp Blood kit (Qiagen, Hilden, Germany) and stored at −80 °C until analysis. The CYP3A5*3 allele was detected using a PCR-restriction fragment length polymorphism (RFLP) method [20]. The results obtained from PCR–RFLP analyses were confirmed using a fully automated SNP detection system (prototype i-density™; ARKRAY, Kyoto, Japan). The results of *CYP3A5* genotyping were not used to adjust Tac-QD dosage or to score acute GVHD.

### Blood concentration of tacrolimus

Whole blood samples were collected with EDTA just prior to the morning administration of Tac-QD 4–7 days after switching from continuous infusion to Tac-QD. Also, samples were collected 4–7 days after switching from intravenous micafungin to oral AZ. During both periods, whole blood tacrolimus concentrations were measured every day to confirm the stability of tacrolimus trough concentrations. Whole blood tacrolimus concentrations were determined using CMIA on the Architect-i1000^®^ system (Abbott Laboratories, Abbott Park, IL) according to the manufacturer’s instructions. The Architect tacrolimus assay is a semiautomated, robust and highly sensitive immunoassay. It represents an alternative approach for laboratories that are not equipped with an LC-MSMS, and it meets the 1 ng/mL LOQ recommendation of the European Consensus Conference on Tacrolimus Optimization [29]. LOQ values for CMIA are reported to be 0.5–0.8 ng/mL, and CMIA exhibits cross-reactivity of 94 % to the tacrolimus active metabolite, 31-*O*-demethyl tacrolimus (M-II) [29, 30]. The reliable range of determination of tacrolimus with the Architect-i1000^®^ instrument is between 0.5 and 30 ng/mL [31, 32].

### Definition of acute kidney injury

Acute kidney injury (AKI) was defined according to CTCAE version 4.0: serum creatinine increased 0.3 mg/dL or more, or more than 1.5 times from the baseline of serum creatinine. Baseline serum creatinine was the value obtained before the start of conditioning therapy.

### Statistical analyses

The primary endpoint of this study was the analysis of the pharmacokinetic behavior of Tac-QD based on *CYP3A5* polymorphisms. The secondary endpoints were the assessments of the development of acute GVHD and the transplantation-related mortality on day 100. The Kolmogorov–Smirnov test was used to assess distribution. The clinical characteristics of the HSCT patients, dose-adjusted blood concentrations and changes in these parameters were expressed as medians (quartiles 1–3). The Chi-square test was used to examine differences in categorical data, except when the expected number of cells was <5, in which case the Fisher’s exact test was used. The Mann–Whitney *U* test was used to determine the significance of difference between continuous values between the groups. The Wilcoxon paired signed-rank test was used to determine the significance of differences in continuous values within each patient. A receiver operating characteristic (ROC) curve was used to determine the best cutoff values for predictive factors that had a minimum distance from the upper left corner to a point on the ROC curve. The proportion of patients showing no clinical events was estimated using the Kaplan–Meier method. The time to clinical events was compared between the groups using the stratified log-rank test. A *P* value <0.05 was considered to be statistically significant. For post hoc power analysis, an effect size was calculated in comparison with clinical characteristics between recipients with or without AKI after co-administration of AZs. An effect size >0.5 was considered clinically meaningful. Statistical analyses were performed using SPSS version 20.0 software for Windows (SPSS IBM Japan, Tokyo, Japan). Power was calculated using G*Power version 3.1 software.

## Results

### CYP3A5 genetic polymorphism and outcome after HSCT

The characteristics of patients are given in Table 1. The *CYP3A5* genotype frequency was in Hardy–Weinberg equilibrium [19]. Eleven patients had either the CYP3A5*1/*1 or *1/*3 genotype, and 13 patients had the CYP3A5*3/*3 genotype. There was no significant difference in the ages, body weights, underlying diseases, pre-HSCT disease status, graft sources, extents of HLA allele matching or conditioning regimens between the two groups. Three patients could not switch to oral AZ due to severe infection. Fluconazole was used in 15 of 21 patients who were given oral AZ. Three patients received itraconazole, whereas another 3 patients were treated with voriconazole.

**Table 1:** Characteristics of HSCT recipients

Characteristics	*CYP3A5*	*CYP3A5*	*P* value
	*1/*1 or *1/*3	*3/*3	
Number of recipients	11	13	
Male/female	8/3	9/4	
Age [years, median (range)]	39 (21–63)	55 (36–65)	0.124
Body weight [kg, median (range)]	51 (49–66)	61 (49–69)	0.825
Diagnosis			0.622
AML/MDS	8	11	
ALL	3	2	
State before HSCT			0.265
CR1	2	4	
CR2 or CR3	3	5	
Non-remission	6	4	
Graft source			0.247
BM from unrelated donors	10	8	
PBSC from sibling donors	0	2	
Umbilical cord blood	1	3	
HLA allele matching			0.543
5/8	1	2	
6/8	2	0	
7/8	2	7	
8/8	6	4	
Conditioning			0.271
Reduced intensity	3	7	
Myeloablative	8	6	
GVHD prophylaxis			0.397
MTX	11	11	
MMF	0	1	
MTX + MMF	0	1	
In combination with AZ			0.855
Fluconazole	7	8	
Itraconazole	1	2	
Voriconazole	1	2	

*AML* acute myeloid leukemia, *MDS* myelodysplastic syndrome, *ALL* acute lymphoblastic leukemia, *HSCT* hematopoietic stem cell transplantation, *CR* complete remission, *BM* bone marrow, *PBSC* peripheral blood stem cell, *HLA* human leukocyte antigen, *GVHD* graft-versus-host disease, *MTX* methotrexate, *MMF* mycophenolate mofetil, *AZ* azole antifungal agent

There was no transplantation-related mortality on day 100 in the present study cohort (Table 2). Two patients with relapse of the underlying disease and two patients with fungal infection were noted. There was a significant difference in the cumulative incidence of grade III–IV severe acute GVHD between the patients with the CYP3A5*1 allele and the CYP3A5*3/*3 allele (36 vs. 0 %, *P* = 0.017). The incidence of AKI was higher in the CYP3A5*3/*3 group than in the *1 allele group (46 vs. 9 %, respectively, *P* = 0.046).

**Table 2:** Acute GVHD, TRM, relapse, fungal infection and AKI during the first 100 days

*CYP3A5* genotype	*1/*1 or *1/*3 (*n* = 11)	*3/*3 (*n* = 13)	*P* value
Acute GVHD			0.173
Grade 0	5	8	
Grade 1	1	1	
Grade 2	1	4	
Grade 3	3	0	
Grade 4	1	0	
Acute GVHD			0.017
Grade 0–2	7	13	
Grade 3–4	4	0	
TRM	0	0	
Relapse	1	1	0.902
Fungal infection	1	1	0.902
AKI	1	6	0.046

*GVHD* graft-versus-host disease, *TRM* transplantation-related-mortality, *AKI* acute kidney injury

### Pharmacokinetics of Tac-QD during administration of oral AZ

**Fig. 1:**
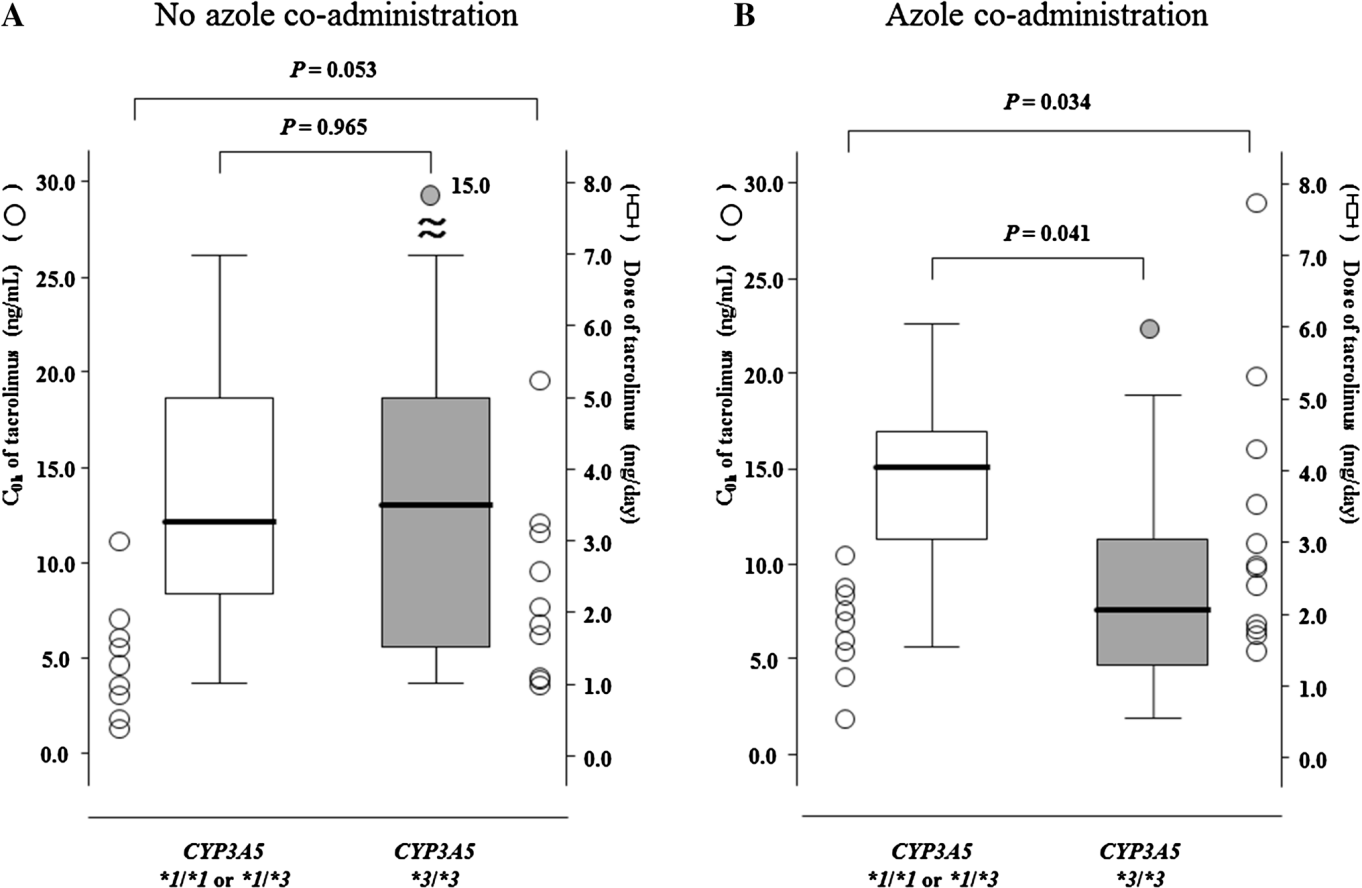
Comparison of doses (*box* and *whiskers plots*) and the tacrolimus trough levels (*open circles*) between the CYP3A5*1*1 + *1/*3 group and the *3/*3 group. **a** Before co-administration of AZ and **b** after co-administration of AZ. Graphical analysis was performed using an SPSS *box* and *whiskers plot*. The *box* spans data between two quartiles (IQR), with the median represented as a *bold horizontal line*. The ends of the *whiskers* (*vertical lines*) represent the smallest and largest values that were not outliers. The *gray circles* represent the outlier of dose. *AZ* azole antifungal agent

Without co-administration of oral AZ, neither the tacrolimus C_0_ nor the median daily dose of tacrolimus differed between the CYP3A5*3/*3 and the CYP3A5*1 allele groups (3.5 vs. 3.3 mg/day, respectively, *P* = 0.965 or 7.9 vs. 4.9 ng/mL, *P* = 0.053) (Fig. 1a). On the other hand, in the presence of AZ, tacrolimus C_0_ values were higher in patients with the CYP3A5*3/*3 than with the CYP3A5*1 allele (10.1 vs. 7.4 ng/mL, respectively, *P* = 0.034) (Fig. 1b). The daily dose of tacrolimus was significantly lower in patients with the CYP3A5*3/*3 allele than with the *1 allele (2.0 vs. 4.0 mg/day, respectively, *P* = 0.041).

### AKI occurred in the CYP3A5*3/*3 group after co-administration of AZ

Seven patients developed AKI in the present study. In order to properly study the impact of Tac-QD and co-administration of AZ, we excluded the three patients who did not receive AZ and the one patient who developed AKI prior to the administration of Tac-QD (Table 3). The median tacrolimus C_0_ with administration of AZ in patients with AKI was about twice that of patients without AKI (16.3 vs. 8.6 ng/mL, *P* = 0.020, effect size = 0.517). Furthermore, only the patients with the CYP3A5*3/*3 developed AKI (6/6 vs. 6/14 patients, *P* = 0.024, effect size = 0.535). AKI occurred within 14 days of starting co-administration of AZ (Supplementary Fig. 1).

**Table 3:** Comparison of clinical characteristics between recipients with or without AKI after co-administration of azole antifungal agents

	With AKI	Without AKI	*P* value
Sex			0.613
Male	4	10	
Female	2	4	
CYP3A5 genotypes			0.024
*1/*1 or *1/*3	0	8	
*3/*3	6	6	
Age [years, median (range)]	60 (39–63)	53 (37–61)	0.353
Body weight [kg, median (range)]	55.1 (46.8–63.3)	61.5 (48.6–75.0)	0.097
In combination with			0.239
Fluconazole	4	11	
Itraconazole	2	1	
Voriconazole	0	2	
Dose of tacrolimus (mg/day)	2.0 (1.5–5.0)	3.5 (3.0–4.0)	0.659
Tacrolimus C_0_ (ng/mL)	16.3 (13.4–16.4)	8.6 (6.8–9.1)	0.020

*AKI* acute kidney injury

**Fig. 2:**
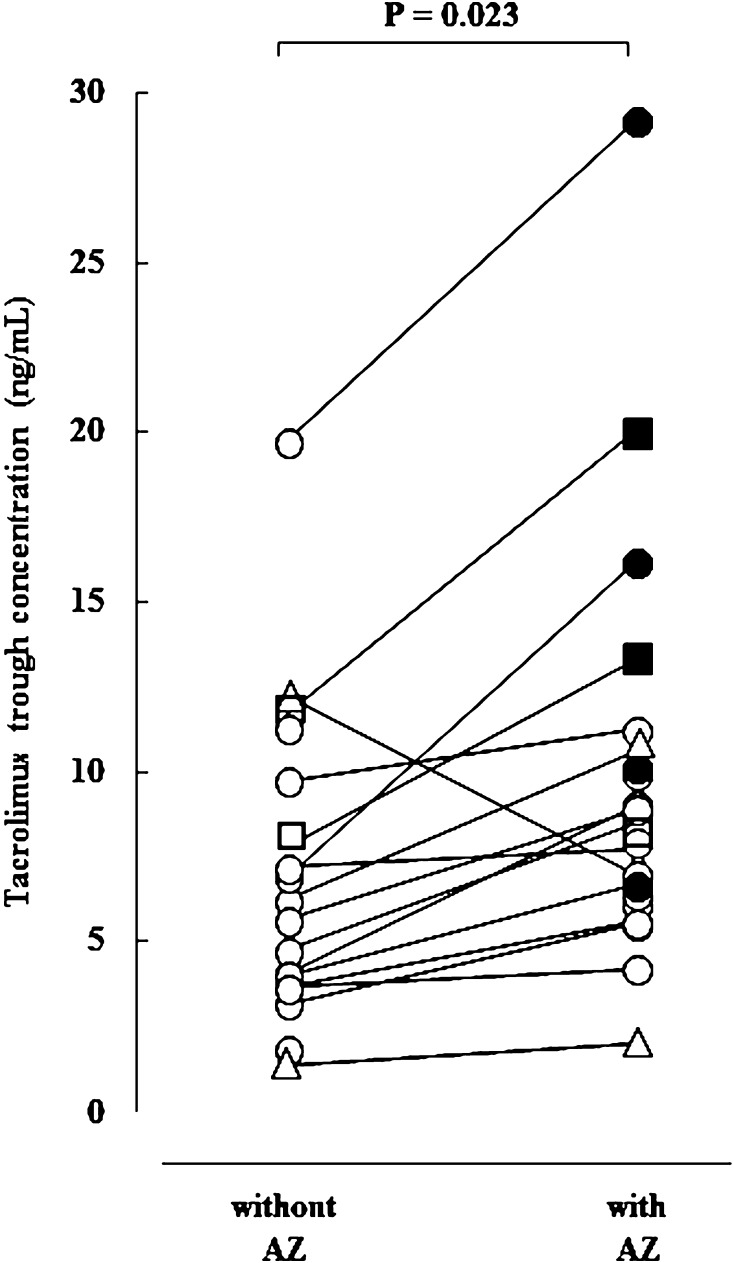
Comparisons of the tacrolimus trough levels according to the presence or absence of a co-administered AZ. *Circles* fluconazole; *triangles* voriconazole; *boxes* itraconazole; *closed figures* patients with AKI. *AZ* azole antifungal agent, *AKI* acute kidney injury

The tacrolimus C_0_ value was higher after co-administration of AZ in most patients than without, and AKI was observed only after co-administration of AZ (Fig. 2). The area under the ROC for the tacrolimus C_0_ to develop AKI after co-administration of AZ was 0.833, which gave the best sensitivity (83.3 %) and specificity (85.7 %) at a tacrolimus C_0_ threshold of 10.1 ng/mL (data not shown).

**Fig. 3:**
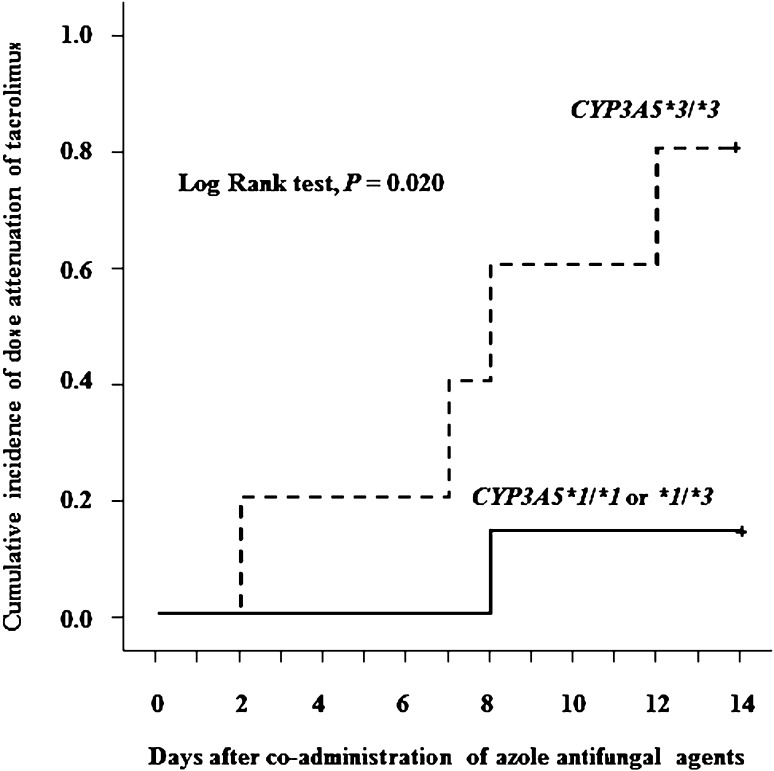
Cumulative incidence of dose attenuation of tacrolimus from baseline (<50 %) after co-administration of AZ. *Solid line*, CYP3A5*1*1 + *1/*3; *dotted line*, CYP3A5*3/*3. Median time to reduction in tacrolimus dosage was 8.0 days in the CYP3A5*3/*3 group and not reached in the CYP3A5*1*1 + *1/*3 group. *AZ* azole antifungal agent

Eighty percent of the CYP3A5*3/*3 group were required to reduce the daily dose of Tac-QD by 50 % or more within 14 days of co-administration of AZ (Fig. 3). The reduction in the Tac-QD dosage in patients with the CYP3A5*3/*3 was significantly faster than those with the CYP3A5*1 allele (*P* = 0.020).

## Discussion

This is the first study to assess the inter-individual variability of tacrolimus blood concentrations in allogeneic HSCT patients who were given Tac-QD. The results were clearly dependent upon the *CYP3A5* polymorphism. There are three clinically important findings in the present study. First, the *CYP3A5* polymorphism had a great influence on the blood concentrations of tacrolimus, even when the once-daily modified-release formulation was used. Second, the blood concentrations of tacrolimus in patients with CYP3A5*3/*3 was elevated markedly after co-administration of AZ, even when the patients had been given fluconazole, which is recognized as having the lowest CYP3A4 inhibitory activity among AZs. Finally, the CYP3A5*3/*3 group with AZ developed AKI when the blood concentrations of tacrolimus were over 10.1 ng/mL.

Our colleagues previously reported that the *CYP3A5* polymorphism had no impact on the dose-adjusted AUC_0–24_ of tacrolimus during continuous intravenous infusion in kidney transplantation patients. However, the bioavailability of oral tacrolimus was higher for the CYP3A5*3/*3 group than for the CYP3A5*1 allele group [33]. Also in the present study, we investigated the pharmacokinetics of tacrolimus when switching from intravenous infusion to oral administration and antifungal agents. AZ has a stronger inhibitory effect on CYP3A4 activity than on CYP3A5 in the small intestine [27, 28], and tacrolimus is metabolized by CYP3A4 in patients expressing CYP3A5*3/*3. As a result of CYP3A4 inhibition with AZ in the small intestine, we found that the C_0_ of tacrolimus was elevated markedly in CYP3A5*3/*3 patients after co-administration of AZ (Fig. 1). It has been reported that the blood concentration of tacrolimus was higher in patients with the CYP3A5*3/*3 than those with the CYP3A5*1 allele in the absence of co-administration of AZ in HSCT and kidney transplantation [17, 34]. We previously confirmed that the influence of CYP3A5 polymorphism on the tacrolimus dosage became evident 14 days after kidney transplantation [35]. Between the two genotypes, we did not observe a significant difference in the C_0_ value of tacrolimus or daily doses of Tac-QD before co-administration of oral AZ. However, it may be too short a time to observe differences in the blood concentration and doses of tacrolimus after switching to oral tacrolimus.

CYP3A5 is expressed in the liver and small intestine [8, 36, 37], and it plays a key role in the small intestine [15]. The interaction between tacrolimus and AZ is depicted in Fig. 3. In patients with the CYP3A5*1 allele, tacrolimus can be metabolized by CYP3A5 in the intestinal epithelium when oral AZ is given, resulting in inhibition of CYP3A4 activity. In contrast, the metabolism of tacrolimus in the CYP3A5*3/*3 group proceeds only through CYP3A4 in the presence of oral AZ. Since HSCT recipients are at high risk of developing invasive fungal infection [23], antifungal agents are commonly used therapeutically and/or prophylactically. Many factors contribute to variability in the clinical significance of drug interactions between tacrolimus and AZ [27, 34, 38, 39]. Although the strong inhibition of CYP3A4 and P-glycoprotein by itraconazole is well known, many reports suggested that the influence of fluconazole on CYP3A4 may be less than other AZ agents [26–28]. In the present study, 15 patients were given fluconazole and eight of them carried the CYP3A5*3/*3. Four patients who were given fluconazole in the CYP3A5*3/*3 group showed nephrotoxicity (Fig. 2). Kuypers et al. [40] reported similar results, in that the CYP3A5*3/*3 patients who were given fluconazole were more frequently exposed to supra-therapeutic tacrolimus C_0_. Our study suggests that co-administration of fluconazole with Tac-QD in the CYP3A5*3/*3 may cause not only elevation of tacrolimus blood concentrations but also kidney injury.

Tacrolimus has a narrow therapeutic window in HSCT, and Yano et al. [41] reported that the modification of Tac-QD to maintain a whole tacrolimus C_0_ above 7.5 ng/mL may be as effective as Tac-BID, and no patient developed grade III–IV acute GVHD. On the other hand, we demonstrated that nephrotoxicity of tacrolimus may increase when tacrolimus C_0_ was above 10.1 ng/mL (Fig. 2). By maintaining the tacrolimus C_0_ under 10 ng/mL, we may prevent nephrotoxicity in patients who are given Tac-QD with AZ. As Ram et al. reported [1], a higher mean blood concentration of tacrolimus during the second week following HSCT was also correlated with protection against grade III–IV acute GVHD (Supplementary Fig. 2). The number of subjects was quite small, and we could not identify the clinical significance of the blood concentration of tacrolimus and *CYP3A5* genotype. Studies consisting of a large number of subjects will be needed.

Unlike kidney transplantation, the administration of antifungal agents is usually required for allogeneic HSCT. *CYP3A5* genotyping may be useful for determination of the appropriate dose of Tac-QD and the selection of AZ, in order to avoid AKI as well as severe acute GVHD. We need further prospective study to determine whether appropriate therapeutic drug monitoring based on stratified doses of tacrolimus in the setting of *CYP3A5* polymorphism can prevent AKI.

## Electronic supplementary material

Below is the link to the electronic supplementary material.

Supplementary Fig. 1. Cumulative incidence of AKI after co-administration of AZ in the CYP3A5*1*1 + *1/*3 group and the *3/*3 group with AZ. Solid line, the CYP3A5*1*1 + *1/*3 group; dotted line, the CYP3A5*3/*3 group. AZ: azole antifungal agent, AKI: acute kidney injury (TIFF 25 kb)

Supplementary Fig. 2. Cumulative incidence of grade III–IV GVHD after hematopoietic stem cell transplantation in the CYP3A5*1*1 + *1/*3 group and the *3/*3 group with AZ. Solid line, the CYP3A5*1*1 + *1/*3 group; dotted line, the CYP3A5*3/*3 group. GVHD: graft-versus-host disease (TIFF 41 kb)
