# Supplementary figures and images for: Effects of CYP3A5 polymorphism on the pharmacokinetics of a once-daily modified-release tacrolimus formulation and acute kidney injury in hematopoietic stem cell transplantation
Source: Cancer Chemother Pharmacol. 2016 May 23;78:111–8. doi: 10.1007/s00280-016-3060-4 (PMC4921119; doi:10.1007/s00280-016-3060-4)

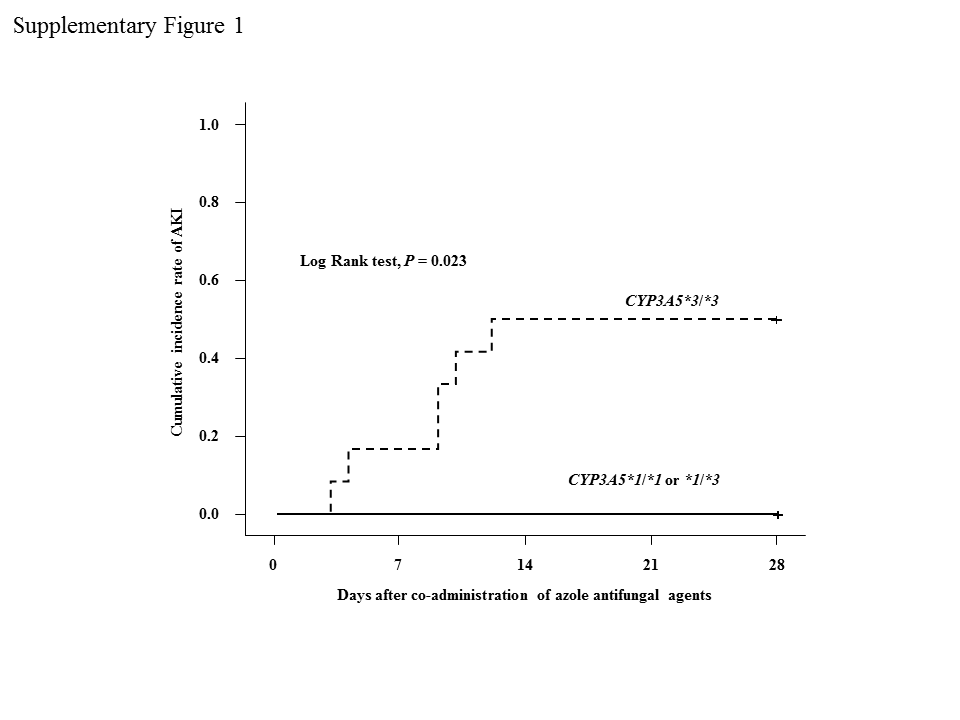

Supplement: Supplementary file 1 — Supplementary Fig. 1. Cumulative incidence of AKI after co-administration of AZ in the CYP3A5*1*1 + *1/*3 group and the *3/*3 group with AZ. Solid line, the CYP3A5*1*1 + *1/*3 group; dotted line, the CYP3A5*3/*3 group. AZ: azole antifungal agent, AKI: acute kidney injury (TIFF 25 kb) [file 280_2016_3060_MOESM1_ESM.tif]

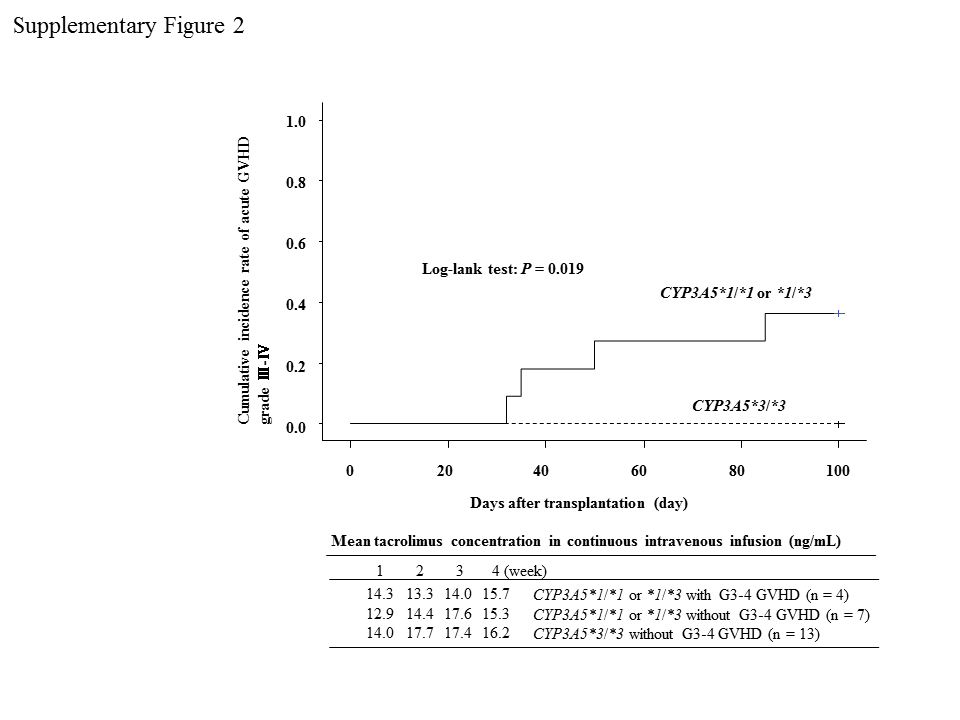

Supplement: Supplementary file 2 — Supplementary Fig. 2. Cumulative incidence of grade III–IV GVHD after hematopoietic stem cell transplantation in the CYP3A5*1*1 + *1/*3 group and the *3/*3 group with AZ. Solid line, the CYP3A5*1*1 + *1/*3 group; dotted line, the CYP3A5*3/*3 group. GVHD: graft-versus-host disease (TIFF 41 kb) [file 280_2016_3060_MOESM2_ESM.tif]
